# Supplementary material for: Probiotics Dietary Supplementation for Modulating Endocrine and Fertility Microbiota Dysbiosis
Source: Nutrients. 2020 Mar 13;12(3):757. doi: 10.3390/nu12030757 (PMC7146451; doi:10.3390/nu12030757)
Supplement: Supplementary file 1 [file nutrients-12-00757-s001.pdf]

Supplementary Table 1. Summary of the effects of probiotics on animal's fertility disorders and their outcomes.

Reference	Population characteristics	Probiotic strain	Doses and Administration pattern	Period of intervention (Weeks)	Disease	Results: Clinical parameters variability
Lagenaur et al. (2011)	4 *Macaca mulatta*	*Lactobacillus jensenii* 1153-1646	Group 1: 1×10^9 CFU/day by intragastric gavage<br>Group 2: 2×10^8 CFU/day by food	Group 1: 1<br>Group 2:3- 4	Intestinal barrier	The gastrointestinal tract serves as a reservoir for lactobacilli that colonize the vagina. Vaginal colonization may be achieved in humans by oral delivery methods.
Kiess et al. (2016)	30 Roosters	*Lactobacillus acidophilus* ATCC 314	1 x 10^7 CFU/day	2	Sperm quality	If Lactobacilli reaches high enough concentrations in the cloaca, then sperm quality may be impacted which could lead to poor fertility.
dos Santos et al. (2018)	42 Roosters	*Bacillus subtilis* QST 713	4.5×10^4 CFU/g of feed ad libitum	4	Sperm quality	There are no negative effects on fertility.
Valcarce et al. (2019)	12 male *Danio rerio*	*Lactobacillus rhamnosus* CECT8361 y *Bifidobacterium longum* CECT7347	1 x 10^9 CFU/day	3	Sperm quality	Increased sperm concentration, total motility, progressive motility, and fast spermatozoa subpopulations. Also, showed different behavior patterns indicating a lower stress-like conduct.
Itoh et al. (2011)	28 BALB/C Mice	*L. gasseri* OLL2809 (heat-killed)	10 x 10^8 CFU/day	3	Endometriosis	Suppression development of endometriosis via activation of NK cells.
Qin et al. (2013)	*Danio rerio*	*Lactobacillus rhamnosus* CICC 6141 // *Lactobacillus casei* BL23	1×10^8 cells/g of basal diet, twice per day	4	Reproductive process	Stimulates (either separately or in combination) follicle maturation, enhance fecundity, and improve egg quality in zebrafish.
Takahashi et al. (2007)	6 Piglets	*Lactobacillus plantarum* Lq80	1 x 10^{10} CFU/day	2	Microbiota dysbiosis	Stimulates the growth of lactobacilli.

Treven et al. (2015)	45 FVB/NHanHsd Mice	*Lactobacillus gasseri* K7 // *Lactobacillus rhamnosus* GG	3.6 × 10^8 CFU/day // 4.1 × 10^8 CFU/day	1	Microbiota and translocation	Both probiotics can modulate the bacterial composition of the microbiota in mesenteric lymph nodes and mammary gland so that it could improve the health of the mammary gland and, ultimately, the health of the newborn.
de Andrés et al. (2017)	11 Balb/C Mice	*Lactococcus lactis* MG1614 // *Lactobacillus salivarius* PS2	~ 10^9 CFU/day	~3	Translocation pathways	Physiological translocation of maternal bacteria during pregnancy and lactation may contribute to the composition of the mammary and milk microbiota.

Supplementary Table 2. Summary of the effects of probiotics founds on systematics reviews.

Falagas et al. (2007)	42 Healthy women	L. rhamnosus GR-1 y L. fermentum RC-14	Grupo 1: 8 × 108 CFU/day<br>Grupo 2: 1.6 × 109 CFU/day<br>Grupo 3: 6 × 109 CFU/day	4	Vaginal dysbiosis	This study confirms the potential efficacy of orally administered lactobacilli as a means to restore and maintain a normal urogenital microbiota.
	64 Healthy women	L. rhamnosus GR-1 y L. fermentum RC-15	>109 CFU/day	8	Vaginal dysbiosis	The combination probiotics is safe for daily use in healthy women, and also it can reduce colonization of the vagina by possible pathogenic bacteria and yeasts.
	59 Premenopausal women	L. rhamnosus GR-1 y L. fermentum RC-16	>109 CFU/day	8	Vaginal dysbiosis	Improvement of the vaginal microbiota towards intermediate or normal Nugent scores.
